# The Oxford Plan for Devonshire?

**Published:** 1921-08-13

**Authors:** 


### The Oxford Plan for Devonshire?

At last there is a general movement throughout the hospital world, as is evident from the fact that the same schemes are being actively discussed in widely different parts of the country. The Oxford and the Sussex schemes, as we all know, have found their way to London. They are also being discussed at Exeter, where an autumn campaign is to be started in October. The Royal Devon and Exeter Hospital, of which Brigadier-General Stirling, C.B., C.M.G., is President, is somewhat better placed financially than it was a year ago, because of the income derived from patients' payments; but it has a debt of £2,122 on the accounts of 1920, and is in need of a bungalow ward for the open-air treatment of children, which will cost £2,500. The immediate plan is to hold a grand bazaar on October 27 and 28 in the new wing, which will probably be finished by the end of the year. All the county towns will be invited to co-operate on behalf of the hospital. The Hon. Treasurer, Major Townshend, has stated that, though the workmen's contributions had been maintained notwithstanding the recent spread of unemployment, an additional revenue of £200 a month was required to balance the accounts.

The President, at the recent quarterly meeting, spoke in favour of the Oxford plan. If the Radcliffe Infirmary, Oxford, had gained 35,000 subscribers to its scheme, surely they could gain 20,000 in Devonshire, a number which, on the same basis of 2d. a week, would mean £8,500 a year. Mr. Walton Turner remarked that Devonshire was larger and more populous than Oxford; and Mr. C. E. Bell made the interesting assertion that the Oxford scheme had been in operation in Herefordshire for twenty years. Is this an allusion to a penny fund connected with the hospital?

The sense of the meeting was clearly in favour of some such scheme as the Oxford scheme being adopted, and a desire was shown not to rely upon the Government grant, but if possible to make the hospital independent by support from within the county. It is felt that this will be forthcoming if a scheme is started which offers some benefit in return for a weekly contribution.

